# Determinants for improving respiratory muscle training after spinal cord injury using real-world clinical data

**DOI:** 10.1038/s41393-026-01242-w

**Published:** 2026-07-14

**Authors:** Gabi Mueller, Marianne Tanner, Amelie Gruber, Sabrina Alessandri, Claudio Perret, Andreas Limacher

**Affiliations:** 1 Cardiometabolic and Respiratory Research, Swiss Paraplegic Research, Nottwil, Switzerland; 2 Faculty of Health Sciences and Medicine, University of Lucerne, Lucerne, Switzerland; 3 Physiotherapy, Swiss Paraplegic Centre, Nottwil, Switzerland; 4 Neuro-Musculoskeletal Functioning and Mobility, Swiss Paraplegic Research, Nottwil, Switzerland; 5 Functioning Information Reference Lab, Swiss Paraplegic Research, Nottwil, Switzerland

**Keywords:** Health occupations, Health care

## Abstract

**Study Design:**

Single-center, retrospective analysis of real-world clinical data.

**Objectives:**

To quantify the influence of personal, lesion-, and training-specific parameters on improvements in inspiratory training resistance during a respiratory muscle training period in persons with SCI.

**Setting:**

SCI rehabilitation hospital in Switzerland.

**Methods:**

Men and women with traumatic or non-traumatic SCI, 18 years or older, who performed at least 10 inspiratory training sessions with a preliminary measurement of maximal inspiratory muscle strength (MIP) were included. Inspiratory resistance training was conducted in a group setting of the physiotherapy department. Training prescription: 7 × 10 repetitions per training session at the highest possible intensity, 3–5 times a week during the inpatient rehabilitation stay. Demographic data and training parameters were summarized by median with 25 and 75% percentiles or absolute and relative frequencies. Most important parameters (personal and lesion characteristic as well as training volume and intensity) for improvement of inspiratory training resistance were analyzed by a random forest and an explanatory linear model.

**Results:**

Training intensity and the total number of training sessions have a much greater impact on improvements in inspiratory training resistance than personal or lesion characteristics. Our model showed an adjusted R² of 0.50.

**Conclusions:**

While performing respiratory muscle training in persons with SCI, therapists should focus on a high training intensity, i.e. setting the inspiratory resistance as high as possible, and motivating patients to perform the training for as long as possible, preferably more than 3 months.

**Figure:**
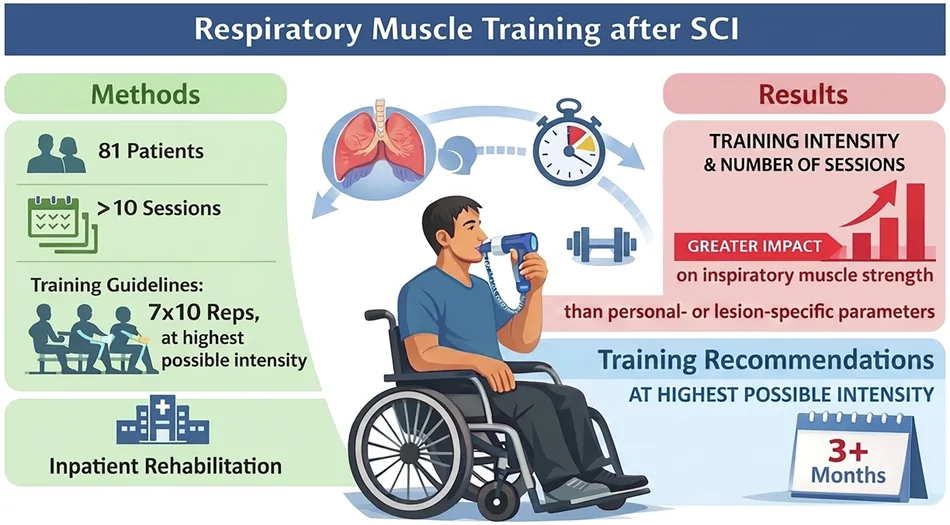

## Introduction

After a spinal cord injury (SCI) respiratory muscle weakness is a common problem, especially in patients with cervical lesions and ASIA impairment scale (AIS) A or B [1–3]. Weak respiratory muscles may lead to complications such as atelectasis or pneumonia [1, 4, 5], which remain among the leading causes of death in individuals with AIS A or B SCI [6]. First evidence shows that inspiratory muscle strength may be an important factor for prevention of respiratory complications such as pneumonia [7]. An increase in maximal inspiratory pressure (MIP) can improve cough capacity [8], resulting in a more effective secretion clearance [4]. In a former study, our group found that patients with MIP values of >90 cmH_2_O, are at substantially lower risk of pneumonia than those with MIP < 90 cmH_2_O [7]. According to a Cochrane review, both in- and expiratory muscle strength can be improved by respiratory muscle training (RMT) in individuals with SCI [2]. Therefore, RMT may, among other interventions, be useful to prevent pneumonia.

RMT is part of the physiotherapy program in many rehabilitation facilities for persons with SCI. However, therapy time, motivation and fatigue of the patients, and finances to cover all the interventions are limited. Therefore, optimal training conduction, with targeted - at best personalized - training prescription is increasingly required to achieve the best possible improvements from invested therapy time. In a recent study conducted by our group [9], we found that training intensity, i.e., the relative training resistance in relation to the individual MIP, is key for improvements in respiratory muscle strength; the higher the intensity, the greater the training improvements.

Nevertheless, individuals with SCI may respond to the same training stimuli with even higher variability than persons without respiratory muscle impairments. Differences in lesion level and severity, which determine innervation of respiratory muscles, may alter the potential for training improvements [1]. In addition, other personal factors, such as sex, age or time post injury may influence training outcomes, i.e. the individual improvement in inspiratory training resistance. A recent systematic review and meta-analysis showed that there is still no consensus on the optimal amount and intensity of respiratory muscle training for best possible outcomes [10].

Therefore, further investigation of personal- and lesion specific parameters on improvement of respiratory muscle performance through analysis of real-world clinical data, may provide further insights into how to best train respiratory muscles in persons with SCI. Ultimately, this could improve cost-effectiveness and potentially increase patient motivation by providing better understanding of the factors that most influence training success.

The objective of this study was to assess which personal- or lesion-characteristics, or training-specific parameters, have the greatest influence on improvements in training resistance. This should help to identify factors for individualized training prescription.

## Methods

### Design and setting

This is a retrospective cohort study of individuals with SCI who performed RMT during inpatient rehabilitation in a stationary setting, embedded into clinical routine and collected between June 2019 and November 2024.

### Statement of ethics and study approval

The research described in this study was conducted in accordance with the World Medical Association Declaration of Helsinki [11]. The study has been approved by the local ethics committee. All participants have given their written informed consent for the retrospective use of their clinically assessed data for research purposes. We certify that all applicable institutional and governmental regulations concerning the ethical use of data of human volunteers were followed during this research.

### Study population

Inclusion criteria were: men and women, 18 years of age or older, traumatic or non-traumatic SCI, participation in the clinical respiratory muscle training group with 10 or more documented respiratory resistance training sessions, MIP measurement before the start of the RMT and a signed general consent for the retrospective use of the data.

Exclusion criteria were: Severe mental disorders, acute or progressive respiratory diseases, progressive neurologic diseases such as multiple sclerosis or amyotrophic lateral sclerosis, Guillain-Barré-syndrome or critical illness polyneuropathy.

### Respiratory function measurements

The measurement of MIP was performed as part of the clinical routine before the start of the RMT, using a respiratory pressure meter (Micro RPM, Micro Medical, Hoechberg, Germany). The respiratory muscle strength assessment followed the American Thoracic Society/European Respiratory Society guidelines [12] and was conducted in a standardized way by specialized healthcare workers from the Respiratory Care Team of the rehabilitation center. For respiratory function measurement, the participants had to breathe through a mouthpiece while wearing a nose clip. After a full expiration, patients had to inhale as strong as possible against a resistance for at least 2 s. MIP measurement was performed under volitional, but standardized instruction, at least three times or until the best three values were within a range of 20%. The highest value (mean pressure over the best second) was entered into the study database and used for analyses.

### Respiratory muscle training

Patients performed the RMT in a supervised group setting of the physiotherapy [13], in a sitting position in their own wheelchair or on a chair (patients with the ability to walk). Five training sessions per week were offered, Mondays to Fridays afternoon, and each patient followed as many sessions as possible, based on their individual therapy schedule. Training prescription was to conduct respiratory muscle training at the highest possible intensity (i.e. inspiratory resistance) which allows to perform about 7 sets of 10 repetitions per training session, for 3–5 times a week. Once a patient was able to perform the training session with less subjective effort than the session before, based on the rating of perceived exertion (RPE) [14], the resistance was increased by the responsible physiotherapist for the next training session. After each training session, the training resistance and count of training repetitions as well as the RPE (6-20) were recorded for each patient. The individual training period was not defined in length, but should contain as many sessions as possible, which is often limited by the duration of the patient’s inpatient rehabilitation stay.

The training devices were small hand-held inspiratory resistance devices with a spring-loaded valve. Based on availability in the clinic and from the supplier, either the POWERbreathe^®^ device (POWERbreathe^®^ International Ltd. Warwickshire, UK) or the PowerLung^®^ device (PowerLung^®,^ Inc., Houston, TX, USA) was used, which are both based on the same principle of resistive loaded breathing. The valve provides an adjustable inspiratory pressure training load, and the patient must generate sufficiently negative pressure to open the inspiratory valve and inhale air. The valve is calibrated and can be adjusted from 4 up to 172 cmH_2_O (POWERbreathe^®)^, and from 4 to 85 cmH_2_O (PowerLung^®^) dependent on the model used.

### Data extraction and storage

All baseline demographic data (sex, age, level and AIS grade of the lesion, weight, height, time post injury and cause of lesion) were extracted from patient’s medical records and stored in the study database. MIP measurements before the start of the RMT were entered into this database as well and all training specific data of each patient (device used, start and end date of the training, total number of training sessions and mean number of training sessions per week, mean number of repetitions per training, initial training pressure, mean training pressure and final training pressure) were calculated at the end of each individual patient’s training journey and added to the study database continuously. Mean training intensity was calculated as the average training resistance over the entire training period, expressed as a percentage of the individual baseline MIP.

### Statistics

The clinical data were imported and analyzed in R (version 4.4.2). Demographic data and training parameters were summarized by median with 25 and 75% percentiles or absolute and relative frequencies.

The variable importance of various covariates (personal, lesion-related, and training parameters) for improvement of inspiratory training resistance was first evaluated using a random forest to inform the development of a parsimonious explanatory model. This non-parametric method can capture linear, non-linear, and interaction effects, and handles collinearity and overfitting effectively. A higher relative incremental mean squared error (%) indicates greater variable importance.

Subsequently, we assessed associations of relevant covariates with inspiratory training resistance improvement using a linear model. The number of training sessions was excluded since the total number of training sessions is already captured by the mean sessions per week and the number of training weeks. Incorporating these two factors into the model added more granularity to key training parameters, so we decided to include them rather than the total number of sessions. Due to low numbers, we categorized AIS grade into A/B vs. C/D. We opted for this distinction because motor function is most relevant for training improvement. In addition, two clinically less relevant variables with low importance in the random forest were omitted (etiology and time since injury) to avoid overfitting, given the sample size of approximately 80 patients (rule of thumb: one factor per 10 patients, i.e. eight factors). Model diagnostics were satisfactory. Normality of residuals and homoscedasticity were evaluated by inspecting Q–Q plots and residual vs. fitted value plots. Model fit was summarized with adjusted R². Potential non-linear quadratic relationships and interactions were examined using a penalized Lasso regression to avoid overfitting, but no biologically plausible non-linear patterns or effect modifications were identified.

We performed additional analyses to exclude any potential influence of the training device or time since injury (persons with acute vs. chronic SCI). To assess the effect of the training device, two variables were included in the model: whether patients started with the PowerLung device (yes vs. no) and whether they switched from POWERbreathe^®^ to the PowerLung^®^ during the course of training (yes vs. no). To further explore any potential influence of time since injury, we conducted subgroup analyses for patients with shorter (≤12 months, n = 62) versus longer time since injury (>12 months, n = 19). Given the small sample size in the latter subgroup, only variables that were significant in the main model were included in this subgroup analysis.

## Results

**Fig. 1:**
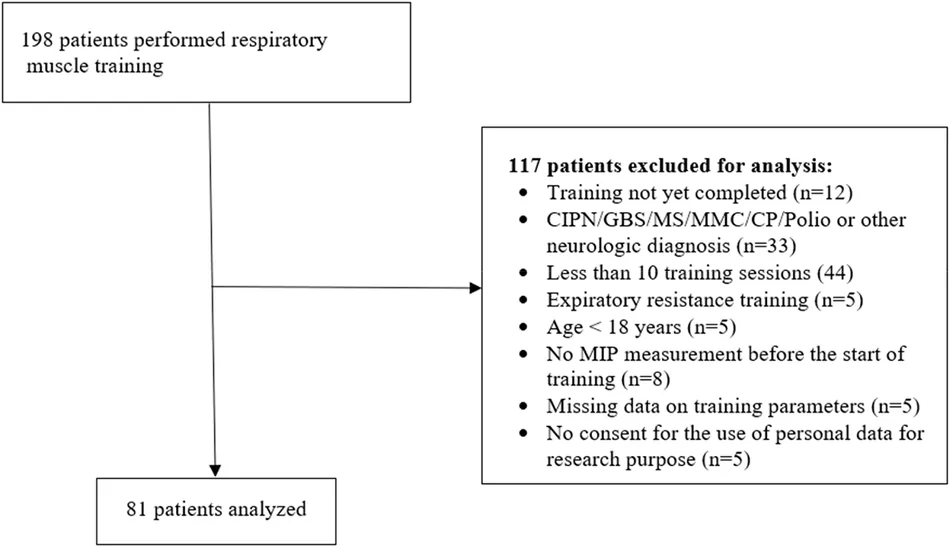
Flow-chart of excluded and analyzed patients. CIPN = Critical Illness Polyneuropathy, GBS = Guillain-Barré Syndrome, MS = Multiple Sclerosis, MMC = Myelomeningocele, CP = Cerebral Palsy, MIP = Maximal Inspiratory Pressure.

In total, 198 persons participated in the RMT group between January 2019 and November 2024. Data from 117 individuals were excluded for the reasons shown in Fig. 1, leaving 81 participants for analysis.

Patient characteristics of the analyzed individuals are presented in Table 1, and their training-specific data are presented in Table 2.

**Table 1:** Characteristics of study participants (no missing values).

Characteristics	Median or N (25, 75% percentiles or %) n = 81
**Age (years)**	65 (54, 73)
**Sex**	
male	59 (73%)
female	22 (27%)
**Lesion level**	
thoracic/lumbar	36 (44%)
cervical	45 (56%)
**AIS**	
A	26 (32%)
B	11 (14%)
C	11 (14%)
D	33 (41%)
**Etiology**	
non-traumatic	27 (33%)
traumatic	54 (67%)
**Time since injury** (mts)	2.7 (1.3, 10.6)

*AIS =* American Spinal Injury Association.

Impairment Scale; mts = months.

**Table 2:** Training-specific parameters (no missing values).

Training parameter	Median (25,75% percentiles) n = 81
**Mean training intensity** (%)	68 (53, 96)
**Number of training sessions**	29 (15, 45)
**Number of training weeks**	10.3 (5.1, 17)
**Mean training sessions per week**	3.2 (2.4, 3.7)
**Mean repetitions per training**	70 (70, 75)
**Inspiratory resistance start** (cmH₂O)	18 (15, 32)
**Inspiratory resistance end** (cmH₂O)	46 (39, 59)
**Inspiratory improvement** (cmH₂O)	26 (14, 38)
**Inspiratory improvement** (%-change)	136 (56, 255)

*cmH₂O =* centimeters of water.

**Fig. 2:**
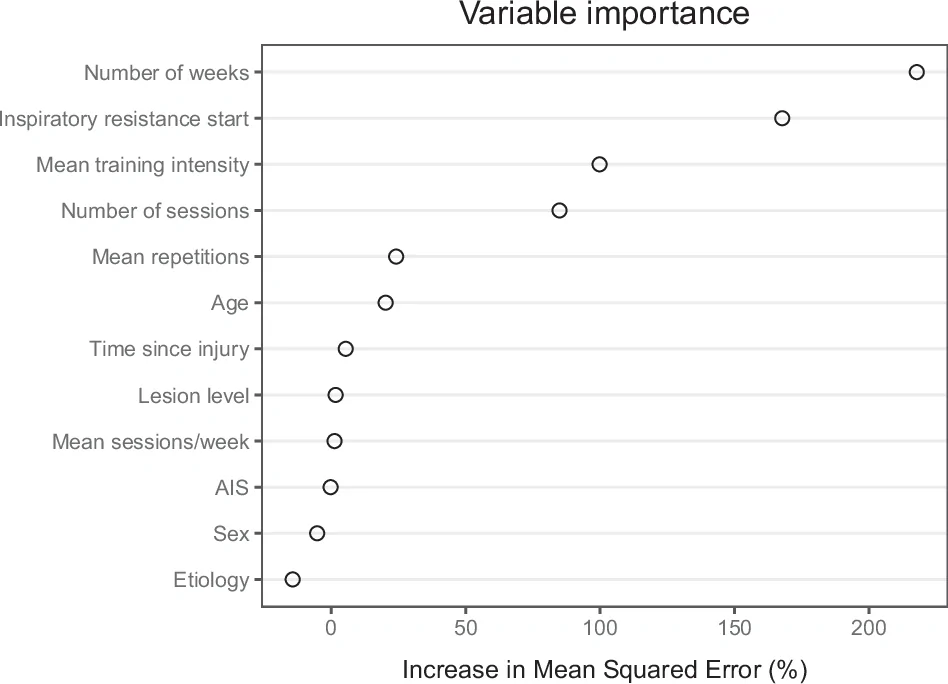
Random forest for inspiratory improvement. Lesion level = C2-C7 vs. T1-L5. AIS = ASIA impairment scale.

The variable importance of various patient characteristics and training parameters using a random forest is shown in Fig. 2. Except for the number of training sessions per week, the most important variables were training-specific parameters – namely, training duration (number of weeks) and intensity – whereas patient and lesion characteristics appeared less important.

Associations between relevant covariates and inspiratory training resistance improvement are shown in Table 3. The modifiable variables with the greatest influence on inspiratory training resistance improvement were training intensity, with an improvement in inspiratory resistance of 0.2 cmH₂O per %-unit increase in training intensity (95% CI 0.1 to 0.3), and the number of training weeks, with an improvement of 1.7 cmH₂O per week of training (95% CI 1.1 to 2.2). This model (Table 3) showed an R² of 0.56 and an adjusted R² of 0.50.

**Table 3:** Explanatory model for inspiratory training pressure improvement.

Variable	Beta	(95% CI)	p-value
**Intercept**	5.9	(−31 to 42)	0.7
**Age** (per year)	−0.07	(−0.33 to 0.18)	0.6
**Gender** (female vs. male)	−5.2	(−14 to 3.5)	0.2
**Lesion level** (C2-C7 vs. T1-L5)	−2.6	(−11 to 5.7)	0.5
**AIS score** (A/B vs. C/D)	2.8	(−5.3 to 11)	0.5
**Inspiratory training resistance start** (per cmH₂O)	−0.52	(−0.85 to −0.19)	0.002*
**Mean training intensity** (per %-unit)	0.20	(0.10 to 0.30)	<0.001*
**Mean training sessions per week** (per session)	5.5	(0.83 to 10)	0.021*
**Mean repetitions per training session** (per repetition)	−0.17	(−0.48 to 0.14)	0.3
**Number of training weeks** (per week)	1.7	(1.1 to 2.2)	<0.001*

*CI* = confidence interval, *cmH₂O* = centimeters of water.

^*^Significant difference between groups; p < 0.05.

In the additional analyses examining the influence of the device used and time since injury, we found no statistical evidence of an effect on the main model. Concerning the use of POWERbreathe^®^ or PowerLung^®^, i.e., whether patients started with PowerLung^®^ (vs. POWERbreathe^®^) and whether patients switched from POWERbreathe^®^ to PowerLung^®^ during the course of training, neither variable showed a significant association with inspiratory improvement, nor did their inclusion substantially affect the estimated effects of other variables (Supplement 1). Concerning time since injury, we found that the estimated effects of model variables were similar across subgroups (acute vs. chronic SCI) and consistent with those observed in the full dataset, suggesting that time since injury does not substantially influence inspiratory improvement or modify the effects of other variables (Supplement 2).

## Discussion

This study examined the impact of personal, lesion-related, and training-specific parameters on improvements in inspiratory training resistance, using embedded clinical research within routine physical therapy practice in an inpatient rehabilitation setting. Training specific parameters such as the number of training weeks and training intensity had a much higher influence on improvements in inspiratory training resistance than personal or lesion-specific parameters. Therefore, we suggest that physiotherapists focus on training at the highest possible intensity for at least three months and perform proper initial and follow-up MIP measurements, to provide optimal individual guidance of the training and to maintain patients’ motivation high.

### Study population

The studied population represents a typical clinical population with SCI, who performed the training based on clinical indication, i.e. a need to increase respiratory muscle strength to prevent secondary health conditions. They had a relatively high median age of about 65 years (Table 1) and should therefore be considered an ageing population. However, evidence from individuals with skeletal muscle function deficits indicates that resistance training is effective and beneficial for strength improvement even with older age [15]. The fact that about three quarts of all participants were male, is normal for persons with SCI and is therefore comparable to other studies in this population [16] and representative for the whole population with SCI.

### Training parameters

The findings of this study clearly show that improvements in training resistance depend not only on high training intensity, but also on longer training duration, i.e. the number of training sessions or weeks While some literature advocates very high training frequencies (e.g. two sessions per day) [17, 18], our study found no significant association between the number of training sessions per week and inspiratory improvement. This may be due to the limited variability of this parameter in our sample, as most participants trained approximately three times per week - a frequency that may already be close to optimal [19]. Nevertheless, our patients trained for a longer period of time and at a higher intensity than reported in most published studies on RMT after SCI, resulting in greater improvements in inspiratory resistance than those summarized in recent systematic reviews [10, 16].

### Influence on inspiratory resistance improvements

The random forest analysis (Fig. 2) showed that training-specific parameters, such as the number of training weeks, the inspiratory training resistance at training start, and the mean training intensity, had a much greater variable importance for inspiratory pressure improvement than personal or lesion parameters such as sex, age, and lesion level. These findings are consistent with the explanatory linear model (Table 3), in which all included training parameters (except mean repetitions per training, likely due to limited variability) showed a significant association with inspiratory pressure improvement, while personal or lesion characteristics did not. Furthermore, we found no evidence that these personal or lesion characteristics, including time since injury, modified the effects of training parameters. This suggests that the identified associations are broadly consistent across the heterogeneous SCI population included in this study. These findings are of high relevance for clinical practice and training guidance of the physiotherapists: training improvements depend mainly on how the training is performed rather than on personal or lesion characteristics. This finding shows that proper and individualized settings of the training device at the highest possible intensity, with progressive increase of resistance, motivation of the patients to perform the training three times per week, and to continue this training for at least three months or even longer, are of high importance for optimal strength improvements.

### Strengths and limitations

The retrospective clinical data used in this study enhances its ecological validity by reflecting a real-world rehabilitation setting. A major strength of this study is its relatively large sample size compared to previous analyses on RMT in persons with SCI [2, 16]. Since our sample size (N = 81) is among the largest in this research area, and RMT data shows some variation in intensity, duration, and other parameters, we were able to build an explanatory model to identify the most important factors for inspiratory training resistance improvement. The model fit as expressed by the adjusted R^2^ was rather high; about 50% of the variance can be explained by this model. Given the multifactorial nature of respiratory rehabilitation after SCI, including individual recovery [20], this model appears to encompass the most important parameters influencing improvements in inspiratory training resistance. Nevertheless, individual biological factors may also contribute to variability in training outcomes, along with psychological and environmental factors, such as patient motivation. Especially during the first months of inpatient rehabilitation after SCI, respiratory muscle strength shows natural recovery and improvements independent of any RMT [21] which cannot be seen in persons with chronic SCI. While most patients included in this study started inspiratory muscle training around three months post injury, the sample contained also some patients with chronic injuries (several years post SCI), when respiratory function predominantly decreases with time and especially with higher age. This may have introduced additional noise into the data, contributing to the unexplained variance in the model. However, when adding time since injury to the model, it was not associated with inspiratory muscle strength improvement, nor did its inclusion affect the estimated effects of other variables.

Another limitation is that more than 50 patients had to be excluded due to missing training or measurement data or having fewer than 10 training sessions. This may have introduced potential bias and is a major issue of embedded research, where therapists are responsible for proper documentation and ensuring that measurements are performed for each patient at the right time. Patients who performed only a low amount of training sessions or were not able to perform proper measurements, may be those who are more severely affected by the lesion, experience further medical complications during inpatient rehabilitation, or have mental problems. This potential bias must be considered.

The use of the two different training devices may have introduced another potential source of bias. However, as shown in Supplement 1, the device used did not show a significant association with inspiratory improvement, nor did its inclusion substantially affect the estimated effects of other variables. Therefore, the device used is unlikely to have affected the effect of training on improvements in inspiratory training resistance.

The findings of this retrospective, single center study, must be interpreted with caution, as they are only representative for the range of data captured in this study, i.e. 2–4 training sessions per week, 40–120% MIP for training intensity and around 70–75 repetitions per training session. They may not be applicable to cases with very low training intensities, up to two training sessions per day, or very few repetitions per training session. It has also to be considered that most of the patients performed the RMT during first inpatient rehabilitation after acute injury. Thus, the findings apply within the observed clinical context and data range.

### Clinical implications

The findings of this study can directly be implemented in daily clinical practice, to further improve the quality of inspiratory muscle training in individuals with SCI. A challenge may be to increase training duration, possibly by starting earlier after injury (as soon as mobilization into the wheelchair and active rehabilitation starts) and by instructing and motivating patients to continue the training after the end of inpatient rehabilitation at home, three times per week. This may make a meaningful difference for long-term prevention of pneumonia, especially in the ageing population. A recent study of our group found a reduction of pneumonia risk of 13% per each 10 cmH_2_O increase in MIP [22] which may be substantial especially for the ageing population with SCI.

Another important aspect of clinical implementation is the training intensity at the start of inspiratory muscle training as well as a continuous increase in training resistance. Regular measurement of MIP during the training journey, e.g. once per month, may further improve motivation and guide optimal training progression. A substantial number of patients may have struggled to reach high training intensity due to fatigue, pain tolerance, or scheduling constraints, as RMT sessions must be performed alongside other essential therapies. Therapists’ awareness and continuous supervision of resistance and progression are also crucial for increasing the proportion of patients training at high intensities.

## Conclusion

We found that training specific parameters, such as intensity and number of training sessions, have a much higher impact on improvements in inspiratory training resistance than personal- or lesion characteristics for individuals with SCI. We therefore suggest focusing on a high training intensity and perform such a training with about 70 repetitions per session and for 3–5 times a week, over at least several months (>3 months) for inpatients with acute SCI, independent of lesion- or personal characteristics.

### Oral presentations of the data

Parts of the data reported in this manuscript were presented at the German speaking medical society for paraplegia annual meeting (DMGP), on May 16^th^ 2025, in Heidelberg, Germany and at the International Spinal Cord Society (ISCoS) annual meeting, on October 9^th^ 2025, in Gothenburg, Sweden.

## Supplementary information

## Data Availability

Data of this study are available from the corresponding author upon reasonable request.
