# Supplementary material for: Determinants for improving respiratory muscle training after spinal cord injury using real-world clinical data
Source: Spinal Cord. 2026 Jul 14;64(9):801–6. doi: 10.1038/s41393-026-01242-w (PMC13538071; doi:10.1038/s41393-026-01242-w)
Supplement: Supplementary file 1 [file 41393_2026_1242_MOESM1_ESM.docx]

**Supplement Nr. 1: Subgroup analysis for device used**

To exclude any potential influence of the training device on the main findings, we performed an additional analysis. Two device-related variables were added to the primary linear model, assessing the associations between relevant covariates and the outcome (improvement in inspiratory training resistance): (1) whether participants started training with the PowerLung device (yes vs. no) and (2) whether they switched from POWERbreathe to PowerLung during the training period (yes vs. no). Neither variable was significantly associated with improvement in inspiratory training resistance. Furthermore, the inclusion of these variables did not materially change the estimated effects of the other covariates relative to the original model (Table 3)

**Supplement Table 1: Explanatory model for inspiratory training pressure improvement including training device type.**

| Variable | Beta | (95% CI*)* | p-value |
| --- | --- | --- | --- |
| Intercept | 7.3 | (-30 to 44) | 0.7 |
| Started with PowerLung (yes/no) | 2.1 | (-10 to 15) | 0.7 |
| Switched to PowerLung (yes/no) | -6.2 | (-23 to 10) | 0.5 |
| Age (per year) | -0.07 | (-0.32 to 0.19) | 0.6 |
| Sex (female vs. male) | -5.9 | (-15 to 3.0) | 0.2 |
| Lesion level (cervical vs. thoracic/lumbar) | -2.8 | (-11 to 5.6) | 0.5 |
| AIS score (A/B vs. C/D) | 2.8 | (-5.5 to 11) | 0.5 |
| Inspiratory training resistance start (per cmH₂O) | -0.49 | (-0.84 to -0.15) | 0.005* |
| Mean training intensity (per %-unit) | 0.20 | (0.09 to 0.30) | <0.001* |
| Mean training sessions per week (per session) | 5.2 | (0.37 to 10) | 0.035* |
| Mean repetitions per training session (per repetition) | -0.19 | (-0.50 to 0.13) | 0.2 |
| Number of training weeks (per week) | 1.7 | (1.1 to 2.3) | <0.001* |

* Significant association with outcome p< 0.05; Abbreviations: CI = confidence interval; cmH₂O = centimeters of water, AIS = ASIA Impairment Scale
