# Supplementary material for: Determinants for improving respiratory muscle training after spinal cord injury using real-world clinical data
Source: Spinal Cord. 2026 Jul 14;64(9):801–6. doi: 10.1038/s41393-026-01242-w (PMC13538071; doi:10.1038/s41393-026-01242-w)
Supplement: Supplementary file 2 [file 41393_2026_1242_MOESM2_ESM.docx]

**Supplement Nr. 2 Subgroup analysis for time since injury**

To further explore any potential influence of time post injury, we conducted subgroup analyses for patients with shorter (≤12 months, n = 62) versus longer time since injury (>12 months, n = 19). Given the small sample size in the latter subgroup, only variables that were significant in the main model were included in this subgroup analysis. The estimated effects of model variables were similar across subgroups and consistent with those observed in the full dataset, suggesting that time since injury does not substantially influence inspiratory improvement or modify the effects of other variables.

**Supplement Table 2: Subgroup analysis of patients with short time to injury**

| **Variable** | **Beta** | **95% CI** | **p-value** |
| --- | --- | --- | --- |
| **Intercept** | 19 | -29, 66 | 0.4 |
| **Age** (per year) | -0.14 | -0.48, 0.19 | 0.4 |
| **Sex** (female vs male) | -7.3 | -18, 3.4 | 0.2 |
| **Lesion level** (cervical vs thoracic/lumbar) | -2.4 | -13, 8.5 | 0.7 |
| **AIS** (A/B vs C/D) | 2.5 | -7.6, 13 | 0.6 |
| **Inspiratory pressure start** (per cmH₂O) | -0.55 | -0.96, -0.15 | 0.008* |
| **Mean training intensity** (per %-unit) | 0.18 | 0.06, 0.31 | 0.004* |
| **Mean training sessions per week** (per session) | 4.0 | -1.8, 9.9 | 0.2 |
| **Mean repetitions per training session** (per repetition) | -0.19 | -0.55, 0.17 | 0.3 |
| **Number of training weeks** (per week) | 1.6 | 0.95, 2.3 | <0.001* |
|  |  |  |  |
| Abbreviations: * Significant association with outcome p< 0.05; Abbreviations: CI = confidence interval; cmH₂O = centimeters of water, AIS = ASIA Impairment Scal | | | |

**Supplement Table 3: Subgroup analysis of patients with long time to injury**

| **Variable** | **Beta** | **95% CI** | **p-value** |
| --- | --- | --- | --- |
| **Intercept** | 7.0 | -38, 52 | 0.7 |
| **Inspiratory pressure start** (per cmH₂O) | -0.65 | -1.3, 0.00 | 0.049* |
| **Mean training intensity** (per %-unit) | 0.23 | -0.04, 0.49 | 0.085 |
| **Mean training sessions per week** (per session) | 3.4 | -5.0, 12 | 0.4 |
| **Number of training weeks** (per week) | -0.19 | -2.6, 2.2 | 0.9 |

Abbreviations: * Significant association with outcome p< 0.05; Abbreviations: CI = confidence interval; cmH₂O = centimeters of water
